# The cardioprotection induced by lipopolysaccharide involves phosphoinositide 3-kinase/Akt and high mobility group box 1 pathways

**DOI:** 10.1016/S1674-8301(10)60045-0

**Published:** 2010-07

**Authors:** Xiang Liu, Yijiang Chen, Yanhu Wu, Tuanzhu Ha, Chuanfu Li

**Affiliations:** a Department of Cardiothoracic Surgery, the First Affiliated Hospital of Nanjing Medical University, Nanjing 210029, Jiangsu Province, China; b Department of Surgery, James H. Quillen College of Medicine, East Tennessee State University, Johnson City, TN 37614, USA

**Keywords:** myocardial ischemia/reperfusion, phosphoinositide 3-kinase/Akt signaling, preconditioning, high mobility group box 1, lipopolysaccharide

## Abstract

**Objective:**

The mechanisms by which lipopolysaccharide (LPS) pretreatment induces cardioprotection following ischaemia/reperfusion (I/R) have not been fully elucidated. We hypothesized that activation of phosphoinositide 3-kinase (PI3K)/Akt and high mobility group box 1 (HMGBx1) signaling plays an important role in LPS-induced cardioprotection.

**Methods:**

In *in vivo* experiments, age- and weight- matched male C57BL/10Sc wild type mice were pretreated with LPS before ligation of the left anterior descending coronary followed by reperfusion. Infarction size was examined by triphenyltetrazolium chloride (TTC) staining. Akt, phospho-Akt, and HMGBx1 were assessed by immunoblotting with appropriate primary antibodies. *In situ* cardiac myocyte apoptosis was examined by the TdT-mediated dUTP nick-end labeling (TUNEL) assay. In an *in vitro* study, rat cardiac myoblasts (H9c2) were subdivided into two groups, and only one was pretreated with LPS. After pretreatment, the cells were transferred into a hypoxic chamber under 0.5% O_2_. Levels of HMGBx1 were assessed by immunoblot.

**Results:**

In the *in vivo* experiment, pretreatment with LPS reduced the at risk infarct size by 70.6% and the left ventricle infarct size by 64.93% respectively. Pretreatment with LPS also reduced cardiac myocytes apoptosis by 39.1% after ischemia and reperfusion. The mechanisms of LPS induced cardioprotection involved increasing PI3K/Akt activity and decreasing expression of HMGBx1. In the *in vitro* study, pretreatment with LPS reduced the level of HMGBx1 in H9c2 cell cytoplasm following hypoxia.

**Conclusion:**

The results suggest that the cardioprotection following I/R induced by LPS pretreatment involves PI3K/Akt and HMGBx1 pathways.

## INTRODUCTION

Immune and inflammatory pathways, initiated by the innate immune system, have been implicated in myocardial ischemia/reperfusion (I/R) injury and congestive heart failure (CHF). However, the molecular mechanisms have not been elucidated. Innate immune recognition is mediated by germ-line-encoded receptors that respond to highly conserved macromolecular structures in pathogens called pathogen associated molecular patterns (PAMPs). Upon PAMPs recognition, Toll-like receptor (TLR) 4 recruits an adapter protein, myeloid differentiation factor 88 (MyD88), to initiate the TLR4/MyD88/NF-κB signaling pathway. The importance of NF-κB activation in myocardial I/R has been well documented[1]–[3]. Evidence that toll-like receptors (TLRs) play an important role in cardiovascular disease has been reviewed recently[4]–[6].

Lipopolysaccharide (LPS), also known as endotoxin, is a highly immunogenic antigen, derived from the outer cell wall of gram-negative bacteria, with the ability to enhance the immune response to soluble antigen. LPS also acts as a ligand of TLR4 to activate the phosphoinositide 3-kinase(PI3K)/Akt pathway[7]. Evidence from several lines of investigation suggests that inflammation stimulated with LPS could be attenuated *via* the PI3K/Akt signaling pathway[8]–[11]. Though LPS is well known for its roles in systemic inflammation and myocardial depression in bacterial sepsis, our data indicated that systemic administration of sublethal doses of LPS protected the myocardium against subsequent I/R injury[12].

The PI3K is a conserved family of signal transduction enzymes that are involved in regulating cellular proliferation and survival[13]. Recent data suggest that the PI3K pathway may play an important role as a negative feedback regulator that limits proinflammatory responses[13],[14]. Guha and Mackman[15] have reported that activation of the PI3K/Akt signaling pathway limited the proinflammatory effects of LPS in cultured monocytes. Fukao and Koyasu[16] have speculated that PI3K may be a negative feedback mechanism preventing excessive innate immune responses.

The PI3K/Akt pathway can be activated by TLRs through a MyD88-independent pathway. Stimulation of TLR2 and TLR4 or interleukin-1 receptor (IL-1R) results in the recruitment of PI3K to the receptors[17], suggesting that stimulation of TLRs not only results in the activation of NF-κB through MyD88-dependent pathways, but also activates PI3K/Akt through an alternative pathway that does not signal through MyD88. Others have reported that low dose LPS preconditioning could protect mesenchymal stem cells (MSCs) from oxidative stress-induced apoptosis and enhance survival of engrafted MSCs *via* activation of PI3K/Akt pathway[18],[19].

High mobility group box 1(HMGBx1) is a nuclear protein that is involved in transcriptional activation and DNA folding[20]. However, in addition to its nuclear role, extracellular HMGBx1 has been shown to be a critical mediator of the innate immune response to infection and injury. HMGBx1 is released from activated macrophages and immunostimulated gut epithelial cells in a delayed manner relative to the secretion of the classical early proinflammatory mediators, suchas tumor necrosis factor (TNF) and IL-1[21],[22]. HMGBx1 is also released from necrotic or damaged cells and serves as a signal for inflammation[23],[24]. HMGBx1 levels are increased by I/R in liver and heart, and activation of innate immune system by HMGBx1 in this context requires TLR4-dependent signaling[25],[26]. Recent evidence has demonstrated that the activation of PI3K/Akt reduced the serum levels of HMGBx1 protein and prevented myocardial cells apoptosis in myocardial I/R[27]. Therefore we speculated that LPS-preconditioning induced cardioprotection against myocardial I/R injury by reducing HMGBx1 through a TLR4/PI3K/Akt-dependent mechanism.

## MATERIALS AND METHODS

### Experimental animals

Age-(8-12 w) and weight-(25-30 g) matched male C57BL/10Sc mice were housed in pathogen-free cages with free access to water and standard rodent chow. The experiments outlined in this manuscript conform to the Guide for the Care and Use of laboratory Animals published by the US National Institutes of Health (NIH Publication No.85-23, revised 1996). All aspects of the animal care and experimental protocols were approved by the ETSU Committee on Animal Care. The mice were assigned to the following five groups: normal mice, sham surgery operation, sham surgery operation and LPS, mice subjected to I/R, mice subjected to I/R and LPS. Animals were pretreated by intraperitoneal (i.p.) injection with 80 µg/kg LPS (0.2 mg/mL, Sigma-Aldrich, Inc., USA) or placebo (PBS) for 1 h prior to induction of ischaemia. Following 45 min of ischaemia, the myocardium was reperfused for 4 h. Staining by triphenyltetrazolium chloride (TTC) on the myocardium was performed after 4 h of reperfusion. The mice were sacrificed by injection of an overdose of ketamine, and the hearts were removed 4 h after reperfusion or sham operation. A single heart tissue section (5 mm) was taken from each heart at the same anatomical location, immersion fixed in 4% buffered paraformaldehyde, and embedded in paraffin for preparation of tissue sections. The remaining heart tissue sections were immediately frozen in liquid nitrogen and stored at -80°C.

### Experimental model of myocardial I/R injury

The mice were anesthetized by isoflurane inhalation and ventilated with room air using a rodent ventilator. After left thoracotomy and exposure of the hearts, the left anterior descending coronary artery (LAD) was ligated with 6-0 silk ligature just proximal to its main branching point. The suture was tied using a “shoestring knot” over a 1 mm polyethylene tube (PE-10) that was left in place during the planned period of ischemia. Myocardial ischemia was confirmed by S-T segment changes and ventricular tachycardia on the electrocardiogram. The ischemic area was readily recognized by a cyanotic appearance and a bulging region. The chest was compressed briefly to expel intrapleural air and closed, leaving one end of the coronary sutures protruding from the chest. After completion of 45 min of occlusion, the coronary artery was reperfused by pulling on the exteriorized suture to release the knot. After 4 h of reperfusion, the mice were sacrificed and the hearts were harvested.

### Determination of infarct size/area at risk

The hearts were removed and perfused with saline on a Langendorff system to wash blood from the coronary vasculature. After the suture around the branch of the coronary artery was tied, the hearts were stained with 1% Evans Blue to determine the risk zone. Each heart was then sliced horizontally to yield 5 slices each approximately 0.2 cm thick. The slices were incubated in 1.5% TTC prepared with 200 mmol/L Tris buffer (pH 7.8) for 15 min at 37°C. Viable myocardium was stained red by TTC, while the necrotic myocardium appeared pale white. The slices were preserved in 10% formaldehyde. The apical side of each slice was imaged and the area of infarction on both sides of each slice was determined by an image analyzer. The area at risk was expressed as a percentage of the left ventricle, and the area of infarction was expressed as a percentage of the area of the tissue at risk.

#### In situ apoptosis assay

Hearts were sliced and embedded in paraffin. Three slides from each block were evaluated for percentage of apoptotic/normal cells (apoptotic index), using the TdT-mediated dUTP nick end labeling assay (TUNEL, Boehringer Mannheim, USA). Four slide fields were randomly examined using a defined rectangular field area with magnification of ×200. One hundred cells were counted in each field. For the negative control of TUNEL, heart tissue sections were incubated with the reaction buffer without terminal transferase. For the positive control of TUNEL, heart tissue sections were incubated with 1 Ag/mL of RNase-free DNase for 10 min at room temperature in order to induce nonspecific breaks in DNA before the TUNEL assay was performed.

### Western blot analysis

Cytoplasmic proteins (80 µg) were mixed with 4×SDS sample buffer, heated at 95 °C for 5 min, and separated by SDS-polyacrylamide (12.5%) gel electrophoresis. The separated proteins were transferred onto Hybond enhanced chemiluminescence membranes (Amersham, Sweden) and then incubated with an appropriate primary antibody (anti-IκBα and anti-p-Akt were from Cell Signaling Technology, USA; anti-Akt and anti-HMGBx1 were from Santa Cruz Biotechnology, USA) in Tris-buffered saline with 0.05% Tween 20 containing 5% nonfat dry milk for 1-2 h at room temperature or 4°C overnight. After they were washed three times in Tris-buffered saline-0.05% Tween 20, the membranes were incubated with peroxidase-conjugated second antibody IgG (Cell Signaling Technology, USA) for 1 h at room temperature. After three washes in PBS, the conjugated peroxidase was visualized by enhanced chemiluminescence according to the manufacturer's instructions. The same membranes were probed with anti-GAPDH (Biodesign, USA) after being washed with stripping buffer (100 mmol/L 2-mercaptoethanol, 2% SDS, 62.5 mmol/L Tris-HCl pH 6.7). The protein signals were quantified by scanning densitometry (Genomic Solutions, USA). The results from each experimental group were expressed as relative integrated intensity compared with normal and sham-operated hearts.

### Cell line and culture

The rat embryonic heart-derived H9c2 cells were obtained from the American Tissue Culture Collection, USA and cultured in RPMI-1640 medium (Life Technologies, USA) supplemented with 1% heat-inactivated fetal bovine serum and 9% newborn calf serum, 2 mmol/L glutamine, and an antibiotic-antimycotic mix in a humidified incubator with 5% CO_2_.

### H9c2 cells exposure to hypoxia experiment

H9c2 (rat cardiac myoblasts) cells were cultured in RPMI-1640 medium with 10% serum at 37°C (5%CO_2_), and sub-cultured in 10 cm dishes with 1×10^6^ cells in 10 mL RPMI-1640 medium with 10% serum at 37 °C. When the cells reached 70%-80% confluence, the cells were subdivided into four groups:normal, normal and LPS, group subjected to I/R, group subjected to I/R and LPS, and LPS was adminstrated at 50 ng/mL. After 24 h pretreatment, the cells were transferred to a hypoxic chamber. Hypoxic conditions were achieved using ProOxC System (Biospherix Ltd., USA). This system includes the ProOx controller and C-Chamber, which reduces the oxygen level to 0.5% within 10 min. H9c2 cells were incubated under hypoxic conditions for 12 h, and 4 h of reperfusion in air containing 5% CO_2_, and were harvested and stored at -80 °C.

### Statistical analysis

All data were expressed as mean±SD. Differences among groups were analyzed by one-way analysis of variance (ANOVA). Student-Newman-Keuls (SNK) or the LSD method was used for multiple comparisons. The *P*-value reported was two-sided and a value of *P* < 0.05 was considered statistically significant. All analyses were performed using the SPSS software (Version 12.0, SPSS Inc., USA).

## RESULTS

### LPS pretreatment reduced infarct size

**Fig. 1:**
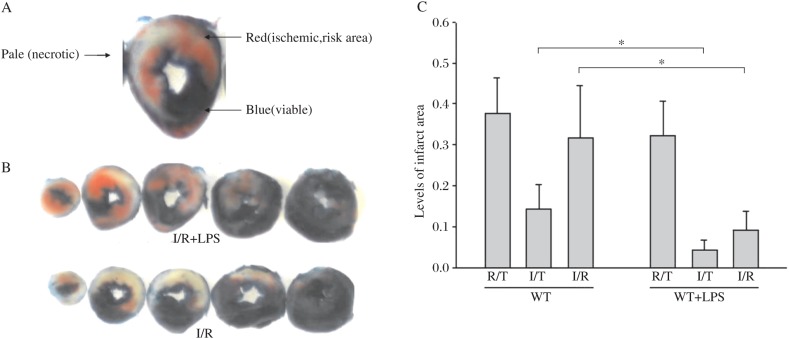
Reduction of infarct size by LPS pretreatment. A: A representative TTC staining of infarct size. Normal myocardium is stained dark blue. The ischemic area is stained red. The infarct area does not stain (pale white). The heart sections shown are from the experiment in A. B and C: LPS reduced myocardial infarction. Wild type (WT) mice were pre-treated with and without LPS (80 µg/kg, i.p. 0.2 mg/mL) for 1 h before the hearts were subjected to 45 min of ischemia followed by reperfusion for 4 h(control *n* = 9; LPS *n* = 8).The infarct size was determined by TTC staining. The areas of the left ventricle (LV)/total(T), area at risk (R), and infarct area (I) were scanned (*P < 0.05).

***Fig. 1A, 1B and 1C*** show that pretreatment with LPS for 1 h reduced the at risk infarct size 70.6% after 45 min ischemia and reperfusion for 4 h (31.62%±4.28%, *n* = 9 *vs* 9.30%±1.58%, *n* = 8) and reduced infarct size of the left ventricle by 64.93% after 45 min ischemia and reperfusion for 4 h (14.24%±2.05%, *n* = 8 *vs* 4.36%±0.86%, *n* = 8), respectively.

### LPS pretreatment decreased cardiac myocytes apoptosis

**Fig. 2:**
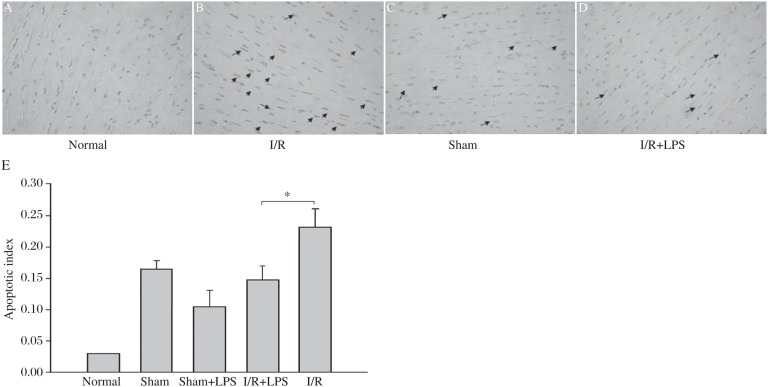
LPS inhibited I/R induced cardiac myocyte apoptosis. LPS (80 µg/kg) was administered for 1 h before the hearts were subjected to I (45 min)/R (4 h). The hearts were sliced, embedded in paraffin, and examined for percentage of apoptotic/normal cells (apoptotic index) by the TUNEL assay. A representative *in situ* TUNEL assay of cardiac myocyte apoptosis is shown [Normal(A); I/R(B); Sham(C); I/R+LPS(D)]. In the TUNEL assay, the blue color shows the nucleus of each cell, and dark brown color indicates positive cardiac myocyte apoptosis. E: Results are expressed as mean±SD of three hearts per group (*P < 0.05).

Cardiac myocyte apoptosis plays a major role in cardiac dysfunction. Therefore, we examined the effects of LPS administration on cardiac myocyte apoptosis in I/R mice. ***Fig. 2A and 2B*** show that pretreatment with LPS for 1h reduced cardiac myocyte apoptosis by 39.1% after 45min ischemia and reperfusion for 4h (23.76%±0.93%, *n* = 3 *vs* 14.35%±1.18%, *n* = 3).

### LPS stimulated the increase in phosphorylation of Akt (p-Akt) in myocardial I/R injury mice

**Fig. 3:**
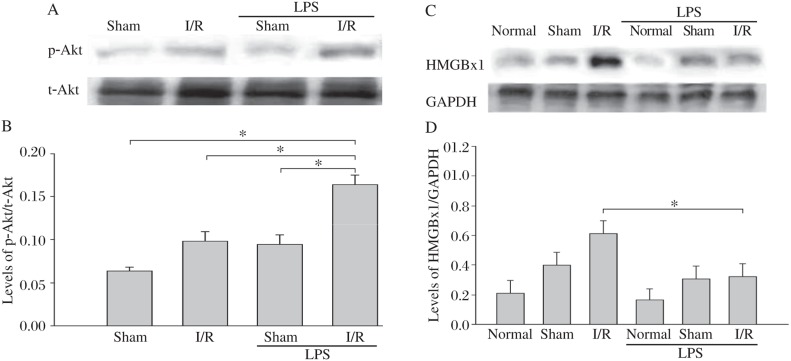
LPS pretreatment stimulated an increase in the p-Akt and a decrease in the HMGBx1 in myocardial of LPS-induced I/R injury mice. LPS was administrated to mice by i.p. injection 1 h before the animals were subjected to I/R. After 4 h of reperfusion, hearts were harvested and cellular proteins were isolated for the examination of the p-Akt (A) and HMGBx1 (C) expression by western blot analysis. There were 4-7 mice in each group. Western blot analysis of p-Akt (B) and HMGBx1 (D) are expressed as means±SD (*P < 0.05).

To examine the effect of LPS on the activation of PI3K/Akt in the myocardial I/R injury mouse model with surgically operated mice serving as sham control, we examined the levels of p-Akt. ***Fig. 3A and 3B*** show that the levels of p-Akt in the myocardium of LPS-pretreated myocardial I/R mice were significantly higher than in the I/R group by 67.5% (16.40%±0.98%, *n* = 4 *vs* 9.79%±0.51%, *n* = 5).

### LPS pretreatment decreased the levels of HMGBx1 in myocardial I/R injury mice

***Fig. 3C and 3D*** show that pretreatment with LPS for 1 h decreased levels of HMGBx1 by 48.30% after 45 min ischemia and reperfusion for 4 h (60.91%±14.89%, *n* = 5 *vs* 31.88%±7.67%, *n* = 5).

### Levels of HMGBx1 increased after hypoxia

**Fig. 4:**
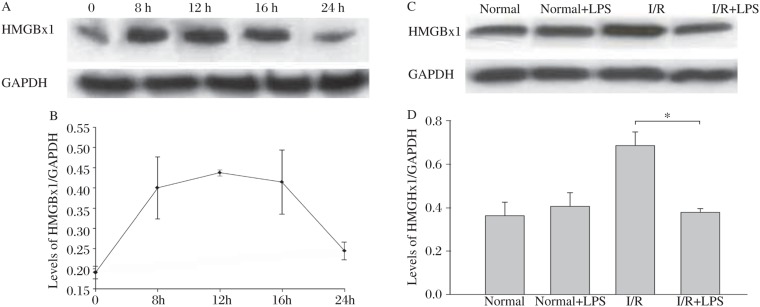
Time points of HMGBx1 expression under hypoxic/reperfusion and effects of LPS pretreatmeat on HMGBx1 level in hypoxic/reperfusion H9c2 cells. A: Western blot results of HMGBx1 at different time points incubated under hypoxic condition for 12h and 4 h of reperfusion. B: The time points of the western blot analysis of HMGBx1. C: Western blot analysis of HMGBx1 detected by specific antibodies Cells were pretreated with LPS at 50 ng/mL. After 24 h pretreatment, cells were incubated under hypoxic conditions for 12 h and 4 h reperfusion in air containing 5% CO_2_. D. Cytoplasmic expression of HMGBx1 in different groups. (*P < 0.05).

***Fig. 4A and 4B*** show that levels of HMGBx1 increased immediately after hypoxia. Levels peaked between 8 to 16 h, and then decreased gradually over the 24 h period. These results suggest that hypoxia induces a rapid but transient increase in HMGBx1 levels and 12 h is an ideal experimental time-point.

### LPS pretreatment reduced the levels of HMGBx1 in hypoxia/reperfusion H9c2 cells

***Fig. 4C and 4D*** show that 12 h hypoxia and 4 h reperfusion induced HMGBx1 cytoplasmic translocation, and levels of HMGBx1 in the cytoplasmic fractions were increased dramatically by 43.2% (68.67%±5.73%, *n* = 3 *vs* 38.02%±4.64%, *n* = 3). Pretreatment with LPS for 24 h reduced levels of HMGBx1 in H9c2 cells by 44.1% after 12 h hypoxia and reperfusion for 4 h (68.67%±5.73%, *n* = 3 *vs* 38.05%±1.53%, *n* = 3).

## DISCUSSION

A novel finding of this study was that a highly purified constituent of the wall of most gram negative bacteria, LPS, reduced myocardial infarction and cardiomyocyte apoptosis in mouse hearts subjected to ischemia (45 min) followed by reperfusion for 4 h. Of potentially greater importance, LPS induced cardioprotection occurred rapidly and did not require a prolonged pretreatment time. Animals were pretreated by i.p. injection with 80 µg/kg LPS for 1 h prior to induction of ischemia.

Our findings suggest that activation of the TLR mediated MyD88-dependent NF-κB pathway may play an important role in myocardial I/R injury, while stimulation of the PI3K/Akt signaling could serve a protective role. The mechanisms of LPS induced cardioprotection involve reducing HMGBx1, while simultaneously activating the PI3K/Akt signaling pathway. In this study, we have observed that hypoxia increased the levels of HMGBx1 in cytoplasm. We found that LPS pretreatment would reduce the levels of HMGBx1 in cytoplasm following hypoxia.

In the previous study, we examined the role of PI3K/Akt signaling in LPS pretreatment induced cardioprotection. We observed that pretreatment of mice with low-dose LPS resulted in significantly increased levels of p-Akt and p-GSK-3β in the myocardium, which positively correlated with cardioprotection. Of greater significance, LPS-induced cardioprotection was abolished in the mice treated with a pharmacological inhibitor of PI3K, as well as in transgenic mice with cardiac specific expression of kinase-defective Akt. Our results indicated that LPS-induced cardioprotection was mediated through a PI3K/Akt-dependent mechanism[12].

Different pharmacological strategies that prevent HMGBx1 secretion (ethyl pyruvate, nicotine, and lysophosphatidylcholine) converge in the inhibition of the NF-κB pathway, and the studies discussed above strongly suggest a link between these two processes. The connection between NF-κB activity and HMGBx1 secretion represents a challenging conundrum, because NF-κB is a transcription factor, and HMGBx1 secretion is not regulated at the level of transcription. Another important consideration is that NF-κB contributes directly to pathologic inflammatory responses, but it also protects parenchyma cells from cytotoxic reagents and hepatocytes from cell death[28]. *In vivo*, inhibition of NF-κB after partial hepatectomy results in massive hepatocyte apoptosis associated with impaired liver function and decreased survival[29]. Moreover, treatment with anti-HMGBx1 antibodies to prevent hepatic injury in response to ischemic insult was associated with enhanced activation of the NF-κB pathway[30]. However, anti-HMGBx1 antibodies abrogated the activation of NF-κB in HMGBx1κB in HMGBx1-challenged enterocytes[31]. This double-edged sword issue makes it challenging to predict the clinical outcome of nonspecific inhibition of NF-κB in human inflammatory diseases and injuries. Unless the therapy is specifically targeted to the control of HMGBx1 secretion from macrophages and monocytes, inhibition of NF-κB activity may not generate an overall beneficial effect, especially in tissue injuries such as acute lung injury or hepatic injury induced by I/R[30],[32]. Future studies will need to determine the molecular links between NF-κB activity and the secretion of HMGBx1 from activated macrophages.

In summary, our data indicate that LPS-induced cardioprotection is mediated, in part, through the PI3K/Akt signaling pathway. These data are significant because they demonstrate that activation of the PI3K/Akt signaling pathway plays an important role in protecting the myocardium from I/R injury by reducing HMGBx1 in LPS-induced cross-tolerance or LPS-preconditioning. Therefore, LPS preconditioning provides a novel strategy in the prevention of the morbidity and mortality associated with myocardial infarction and might be of great benefit for many patients, especially those undergoing cardiac bypass surgery or other procedures in which cardiac arrest is needed.
